# Basosquamous cutaneous carcinoma: a rare case study involving the perineum and review of the literature

**DOI:** 10.1093/jscr/rjae635

**Published:** 2024-10-08

**Authors:** Charles Lu, Mackenzie E Fox, Alexandra F Shapiro, Glenn S Parker

**Affiliations:** Department of Surgery, Hackensack Meridian Health, Jersey Shore University Medical Center, Neptune, NJ, 07753, United States; Department of Surgery, Hackensack Meridian Health, Jersey Shore University Medical Center, Neptune, NJ, 07753, United States; Department of Surgery, Hackensack Meridian Health, Jersey Shore University Medical Center, Neptune, NJ, 07753, United States; Department of Surgery, Hackensack Meridian Health, Jersey Shore University Medical Center, Neptune, NJ, 07753, United States

**Keywords:** basosquamous carcinoma, metatypical carcinoma, basal cell, skin neoplasm, squamous cell

## Abstract

Basosquamous carcinoma (BSC) is a rare form of non-melanoma skin cancer with significant invasive and metastatic potential. This malignancy presents unique challenges in diagnostic and therapeutic options given its ambiguous clinical nature. While there are documented cases of BSC involving the aerodigestive tract and sun-exposed areas like the head and neck, cutaneous BSC in the gluteal and perineal regions remains quite rare. In this report, we provide an overview of the epidemiology, clinical presentation, treatment modalities, and prognosis of this disease. We describe a case of a 71-year-old female who presented for evaluation of a symptomatic right-sided cutaneous lesion in the inner gluteal and perineal region. Upfront surgical resection was pursued for both diagnostic and therapeutic purposes. Final pathology of the lesion revealed infiltrating BSC with negative margins. Although BSC typically presents in sun-exposed areas, the presentation in this patient in a non-sun-exposed area makes this presentation unusual.

## Introduction

Basosquamous carcinoma (BSC) is a rare form of skin cancer prevalent in <2% of nonmelanoma skin cancers, with a preponderance for older Caucasian males commonly located in the head and neck or other sun-exposed areas [1]. It was first described in 1910 and characterized as a variant of basal cell carcinoma that differentiates into squamous cell carcinoma (SCC) [2]. Further studies have demonstrated that BSC features histologic characteristics similar to both basal cell carcinoma and SCC and clinical characteristics similar to SCC, as it has more invasive and metastatic potential [1]. There is also a reported recurrence rate of up to 45% with metastasis in ~5%–10% [1, 3–5]. Although the clinical presentation of BSC may resemble other cutaneous malignancies such as basal cell carcinoma, SCC, or melanoma, helpful dermatoscope findings may include keratin mass, surface scaling, ulceration, white structureless areas, or blood spots on keratin mass [3, 4]. Despite our current knowledge of the clinical and histologic features of BSC, the malignancy remains significantly difficult to diagnose clinically, especially in regions where it is not expected. While dermatoscope results may aid in the diagnosis, the American Academy of Dermatology recommends complete surgical excision as the preferred approach [3, 5]. Alternative options for the removal of BSC include Mohs micrographic surgery, laser ablation, cryotherapy, and chemotherapy [5]. Nonetheless, specific guidelines regarding the management of BSC are lacking. In this report, we present a rare case of BSC involving the perineum in a female patient ultimately treated with surgical resection. We also review the available literature regarding the presentation, diagnosis, and management of BSC.

## Case presentation

We present the case of a 71-year-old female with a past medical history of coronary atherosclerosis, diverticulosis, hemorrhoids, hyperlipidemia, osteoporosis, sleep apnea, and thyroid disease who was evaluated for a cutaneous lesion in the right inner gluteal and perineal region. The patient requested evaluation of the lesion due to discomfort. The patient did not report any associated trauma or systemic signs of disease, such as fever, chills, night sweats, or weight loss. Physical exam findings revealed a hyperpigmented cutaneous lesion on the inner surface of the right gluteus in the perineum, ~1 cm by 0.5 cm, that was well-demarcated and without scaling. The patient had Fitzpatrick skin type of 2, predisposing to an elevated risk of skin cancer [6]. Due to the symptomatic nature of this lesion for this patient and diagnostic uncertainty, surgical excision was pursued. While a dermatoscope may have helped confirm the diagnosis of BSC, upfront surgical resection would achieve both a diagnostic and therapeutic outcome given the symptoms, size, and accessibility of the lesion.

**Figure 1:**
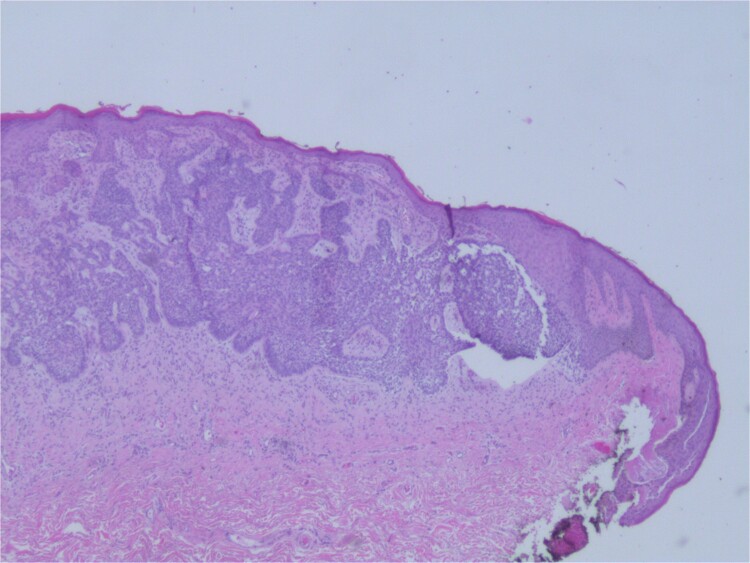
Microphotograph revealing basal cell carcinoma component on histology.

**Figure 2:**
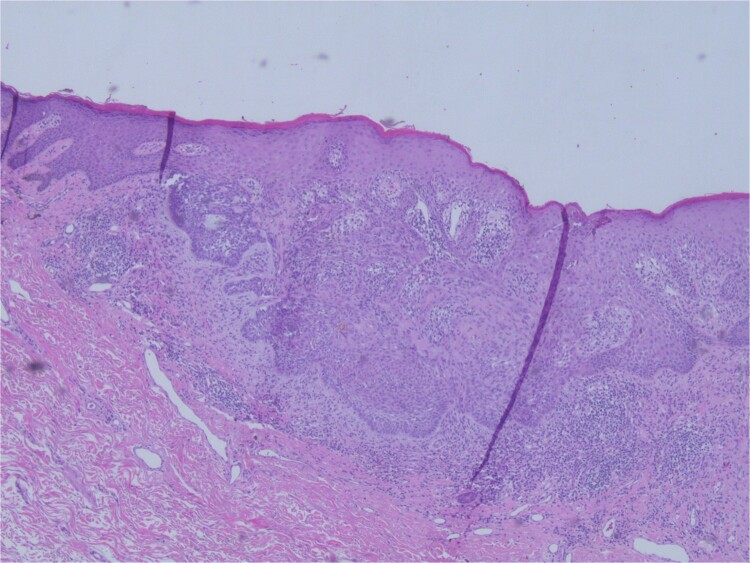
Microphotograph revealing BSC with abnormal squamous keratinization on histology.

**Figure 3:**
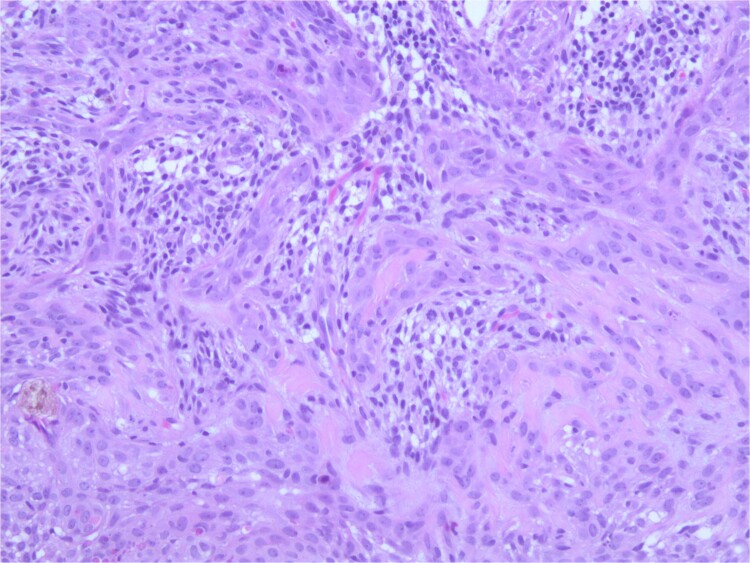
Microphotograph revealing BSC with abnormal squamous keratinization on histology.

**Figure 4:**
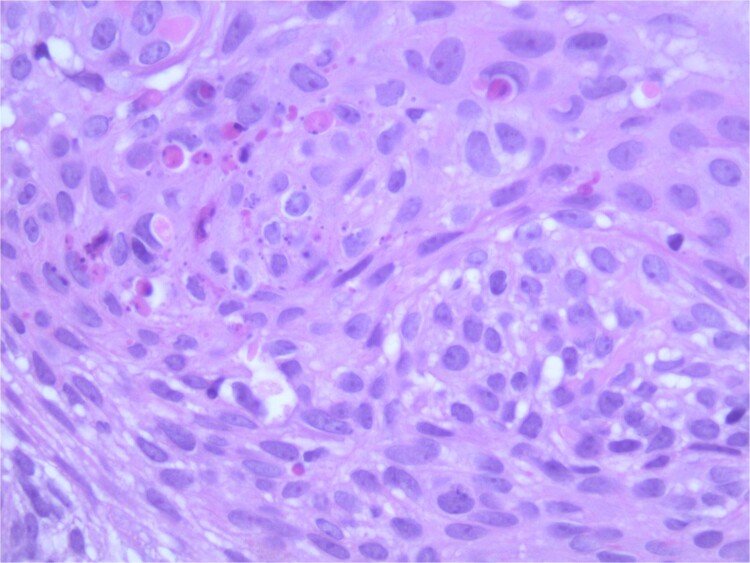
Microphotograph revealing BSC with abnormal squamous keratinization on histology.

At the time of the surgery, the lesion was excised with 5 mm margins, given the acceptable margins of 4 mm for nonmelanoma skin cancer [4]. Macroscopic examination of the lesion revealed a well-defined, hyperpigmented, macular lesion with pathology positive for infiltrating BSC with negative margins confirmed on histology. Histology was notable for predominantly basal cell morphology with areas of squamous differentiation. The presence of the basal cell carcinoma component is demonstrated (Fig. 1). The BSC with abnormal squamous keratinization is also demonstrated (Figs 2–4). These studies were conducted on routine hematoxylin and eosin stains confirming the diagnosis of BSC. The patient was seen in the office, and at 2 months postoperative, there were no clinical concerns.

## Discussion

Basosquamous cell carcinoma, also known as metatypical basal cell carcinoma, is an uncommon type of cutaneous skin carcinoma, and initial presentation in the perineum is extremely rare. The prevalence of this disease is reported to be <2% of cutaneous malignancies [1]. Lack of consensus regarding where BSC falls within the spectrum of basal cell carcinoma and SCC continues to exist; several studies have documented the aggressive behavior of BSC with a metastatic potential similar to SCC [4, 5]. BSC has a predisposition for older Caucasian male patients with cutaneous BSC being more prevalent on the head and neck area (92%) and clinical features that may be nodular, ulcerated, or non-pigmented [3, 4]. These presentations make clinical diagnosis challenging, thus making dermatoscopic analysis and histology via surgical excision important adjuncts. Characteristic histologic features include the presence of a keratin mass, surface scaling, ulceration, white strands or blotches, white structureless areas, or blood spots on keratin mass [4].

Although surgical excision remains the first-line treatment option, there are other treatment modalities, such as Mohs micrographic surgery and radiotherapy [3, 7]. During Mohs micrographic surgery, the malignancy is excised, mapped, and processed into frozen horizontal sections that undergo immediate histologic evaluation. This process is then repeated until histologic evaluation confirms that the malignancy is completely removed [7]. Because BSC is difficult to identify by clinical features alone, patients and providers may elect Mohs surgery in order to preserve the maximal amount of healthy tissue. Radiotherapy is another treatment option and can be considered as an adjuvant treatment when positive surgical margins prevent re-excision of the lesion or when there is local lymph node metastasis. Alternatively, radiotherapy may be considered as an upfront treatment option when surgical excision or Mohs surgery is not achievable. Other treatment modalities include chemotherapy, sonic hedgehog inhibitors, and immune checkpoint inhibitors, which demonstrate varying success [8].

When considering treatment options for BSC, it is important to consider the rate of recurrence and metastasis, as these necessitate more aggressive treatment [1, 4, 5]. Retrospective studies have demonstrated a local recurrence rate between 4% and 47.1% [9] and a metastatic rate of 5%–10% [1, 4, 5]. Factors that increase the rate of local recurrence include positive margins, perineural invasion, and lymphatic invasion [3], and therefore, it is important to ensure negative margins to reduce the risk of metastasis, which makes Mohs and surgical excision effective treatment strategies. Current data regarding the different modalities of treatment also reflects the need for a more established protocol when treating BSC. The National Comprehensive Cancer Network (NCCN) does not, however, provide specific guidelines for the management of BSC [3].

Our patient was treated with surgical excision for both diagnostic and therapeutic indications, which yielded a diagnosis of BSC, an already rare cutaneous malignancy, in an unsuspecting location. This case exemplifies the importance of maintaining a high index of suspicion for cutaneous malignancies in typical and atypical locations.

## Conclusions

BSC, an entity within the spectrum of nonmelanoma skin cancers, shares the features of both basal cell carcinoma and SCC. BSC is difficult to diagnose by clinical exam alone, and surgical excision with 5 mm margins can be an option to achieve both a diagnostic and therapeutic outcome when easily accessible. Although BSC typically presents in sun-exposed areas, the presentation in this patient in a non-sun-exposed area makes this presentation unusual. Long-term follow-up is required.

## Limitations

A limitation of our case report includes short-term follow-up of 2 months. A long-term follow-up would be beneficial in elucidating the sequelae of BSC of the perineum in our patient and associated outcomes. Additional documented cases of rare presentations of BSC may provide further understanding of the disease course and enhance discussions of BSC in the dermatologic and surgical communities.

## References

[1] Martin RC , Edwards MJ, Cawte TG, et al. Basosquamous carcinoma: analysis of prognostic factors influencing recurrence. Cancer 2000;88:1365–9. 10.1002/(SICI)1097-0142(20000315)88:6<1365::AID-CNCR13>3.0.CO;2-Y. 10717618

[2] MacCormac H . The relation rodent ulcer to squamous cell carcinoma of the skin. Arch Middx Hosp 1910;19:172–83.

[3] Ciążyńska M , Sławińska M, Kamińska-Winciorek G, et al. Clinical and epidemiological analysis of basosquamous carcinoma: results of the multicenter study. Sci Rep 2020;10:18475. 10.1038/s41598-020-72732-x. 33116191 PMC7595159

[4] Wermker K , Roknic N, Goessling K, et al. Basosquamous carcinoma of the head and neck: clinical and histologic characteristics and their impact on disease progression. Neoplasia 2015;17:301–5. 10.1016/j.neo.2015.01.007. 25810014 PMC4372646

[5] Garcia C , Poletti E, Crowson AN. Basosquamous carcinoma. J Am Acad Dermatol 2009;60:137–43. 10.1016/j.jaad.2008.09.036. 19103364

[6] Fitzpatrick TB . The validity and practicality of sun-reactive skin types I through VI. Arch Dermatol 1988;124:869–71. 10.1001/archderm.1988.01670060015008. 3377516

[7] Mosterd K , Krekels GA, Nieman FH, et al. Surgical excision versus Mohs' micrographic surgery for primary and recurrent basal-cell carcinoma of the face: a prospective randomised controlled trial with 5-years' follow-up. Lancet Oncol 2008;9:1149–56. 10.1016/S1470-2045(08)70260-2. 19010733

[8] Murgia G , Denaro N, Boggio F, et al. Basosquamous carcinoma: comprehensive clinical and histopathological aspects, novel imaging tools, and therapeutic approaches. Cells 2023;12:2737. 10.3390/cells12232737. 38067165 PMC10706022

[9] Volkenstein S , Wohlschlaeger J, Liebau J, et al. Basosquamous carcinoma-a rare but aggressive skin malignancy. J Plast Reconstr Aesthet Surg 2010;63:e304–6. 10.1016/j.bjps.2009.05.058. 19647505

